# *Escherichia coli* transcriptome assembly from a compendium of RNA-seq data sets

**DOI:** 10.1080/15476286.2023.2189331

**Published:** 2023-03-15

**Authors:** Brian Tjaden

**Affiliations:** Department of Computer Science, Wellesley College, Wellesley, MA, USA

**Keywords:** RNA-seq, transcriptome, bacteria, *Escherichia coli*, compendium, assembly, operons

## Abstract

Owing to the complexities of bacterial RNA biology, the transcriptomes of even the best studied bacteria are not fully understood. To help elucidate the transcriptional landscape of *E. coli*, we compiled a compendium of 3,376 RNA-seq data sets composed of more than 7 trillion sequenced bases, which we evaluate with a transcript assembly pipeline. We report expression profiles for all annotated *E. coli* genes as well as 5,071 other transcripts. Additionally, we observe hundreds of instances of co-transcribed genes that are novel with respect to existing operon databases. By integrating data from a large number of sequencing experiments corresponding to a wide range of conditions, we are able to obtain a comprehensive view of the *E. coli* transcriptome.

## Introduction

The use of RNA-sequencing (RNA-seq) experiments to assay transcript expression has grown rapidly in recent years. As one example, the Sequence Read Archive (SRA), a public repository of next-generation sequencing data, now contains data on more than 25 petabase sequenced pairs from more than 15 million samples [1]. This wealth of data is a terrific resource for transcriptome investigations. However, the management, processing, analysis, and integration of these very large datasets can present challenges. Fortunately, a number of studies have facilitated engagement with large numbers of RNA-seq data sets by providing excellent tools that enable various gene- and transcript- level queries [2–6]. Similarly, computational pipelines have been developed to expedite the processing of large numbers of RNA-seq data sets [7–9]. While transcriptome studies combining hundreds or thousands of RNA-seq samples are common for human data [10–12], there are fewer such studies for bacterial data [13,14].

Large scale bacterial transcriptome studies are important, among other reasons, because the complexity of bacterial RNA biology is still being understood. Post-transcriptional regulation in bacteria is widespread through a variety of RNA elements, such as RNA thermometers [15], riboswitches [16], and small non-coding regulatory RNAs [17]. As one example, small non-coding RNAs in *E. coli* number several hundred, comparable to the number of transcription factors [18]. A first step in understanding the richness of bacterial RNA biology is characterizing bacterial transcriptomes, and RNA-seq experiments are a potent tool for elucidating the full set of transcript expression in an organism.

For *E. coli*, one of the best studied bacteria, a large number of RNA-seq experiments have been performed and there is an abundance of publicly available RNA-seq data. In this study, we use a compendium of 3,376 RNA-seq samples to characterize the transcriptome of *E. coli* K-12 MG1655. We construct transcript assemblies for each sample to evaluate the transcriptional landscape of *E. coli*. We report expression profiles for all annotated *E. coli* genes as well as 5,071 other transcripts that do not correspond to annotated *E. coli* genes, though in general, the other transcripts show significantly less expression than those transcripts corresponding to annotated *E. coli* genes. We also explore the profile of operons in *E. coli* and find nearly three thousand genes being co-transcribed as part of multi-gene transcription units, hundreds of which are likely novel. Using an extensive collection of sequencing experiments based on a diverse set of experimental conditions, we are able to achieve a more complete representation of the *E. coli* transcriptome. To our knowledge, this is the largest transcriptome study to date of a single prokaryotic organism.

## Results

### Dataset

We searched the Sequence Read Archive [1], a public repository of high-throughput sequencing experiments, for RNA-seq samples corresponding to *E. coli* K-12 MG1655. We found 3,376 samples corresponding to 891 different BioProjects. The RNA-seq samples reflect a wide range of scientific studies investigating *E. coli* in varied conditions and conducted by different laboratories. 51 of the 3,376 samples correspond to a size-selection library preparation method. Detailed data for the sequencing read samples are shown at https://dataverse.harvard.edu/api/access/datafile/6562493. FASTQ files containing sequencing information for each sample have a mean size of 7.2 GBs and took 3 minutes to download, on average. In total, it took 171 hours to download the 24 TBs of sequencing data using the SRA Toolkit. The average number of sequencing reads for each sample was 16 million sequencing reads consisting of 2 billion bases. The mean read length was 91 bases with an average quality score of 35. 67% of the samples corresponded to paired, as opposed to single end, reads. In total, the set of sequencing read data contained 52 billion reads consisting of 7.1 trillion bases. Table 1 indicates summary statistics for the compendium of sequencing datasets.

**Table 1.** Statistics summarizing information about the 3,376 RNA-seq samples and their analysis. Information is provided for the average of the 3,376 samples and for the total of all 3,376 samples.

	Average	Total
File size	7.2 GBs	24 TBs
Number of reads	16 million	52 billion
Number of bases	2 billion	7.1 trillion
Read length	91 bases	-
Paired reads	67%	-
Quality score	35	-
FASTQ download time	3 minutes	171 hours
Alignment time	31 minutes	1,775 hours
Sorted binary format time	18 minutes	1,001 hours
Assembly time	8 minutes	455 hours
Alignment rate	76%	-

### Pipeline

**Figure 1.**
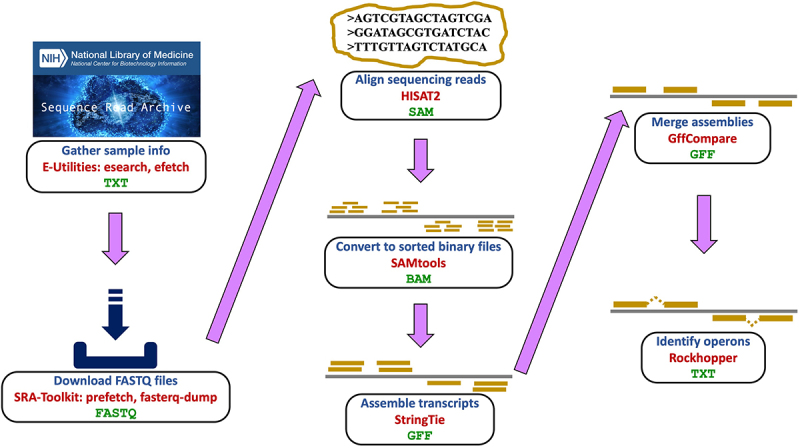
Overview of pipeline for acquiring and evaluating the compendium of RNA-seq data sets for *E. coli*. Data were obtained from the Sequence Read Archive and analyzed by a number of bioinformatic tools in a seven stage process. For each stage depicted, blue text describes the stage, red text specifies the tools used, and green text indicates the resulting file format.

We developed a bioinformatics pipeline for analysis of the massive sequencing dataset (Figure 1). At the core of the pipeline are the tools *HISAT2* and *StringTie* from the popular Tuxedo suite [19], which support sequencing read alignment to a reference genome and transcript assembly, respectively.

The most time consuming component of the pipeline corresponds to aligning the sequencing reads to the reference genome. On average, sequencing reads from a sample took 31 minutes to align to the *E. coli* genome, with 76% of the 52 billion reads successfully aligning with high confidence (Table 1). Overall, the pipeline required 3,402 hours of computation to complete execution. Because samples could be processed independently from one another, we observed an approximately linear speed up in execution time as we increased the number of employed computer processors. Using 24 processors in parallel on a server with 3 GHz Intel Xeon Gold processing, the wall-clock execution time of the pipeline required 359 hours.

### Transcript assembly

As part of the pipeline, RNA-seq alignments were assembled into putative transcripts. Thus, for each sample, a set of assembled transcripts was identified corresponding to transcript expression in the particular experiment. The 3,376 transcript assemblies were then merged using the *GffCompare* tool to establish a single set of transcripts based on expression observed from all samples. Altogether, 9,581 transcripts were identified in the merged assembly: 4,510 transcripts corresponding to annotated *E. coli* genes (https://dataverse.harvard.edu/api/access/datafile/6562494) and 5,071 other transcripts (https://dataverse.harvard.edu/api/access/datafile/6562495). When comparing the merged assembly to annotated *E. coli* genes, the merged assembly has sensitivity of 100% at both the base level and the gene level [20] indicating that transcripts are found spanning the extent of all annotated *E. coli* genes. In addition to transcripts corresponding to annotated genes, 5,071 other transcripts were observed in the 3,376 samples. The extent to which these other transcripts correspond to false-positive transcript identifications in the context of being non-functional remains unclear. These other transcripts may correspond to, for example, transcriptional noise, anti-sense transcription, or other novel transcripts such as yet to be annotated regulatory RNAs or small peptides, and merit further investigation.

In order to evaluate how the choice of computational tools used in the pipeline impacts the results, we employed an alternative tool to *HISAT2* and *StringTie* for aligning reads and assembling transcripts. Using the Rockhopper system [21], we performed sequencing read alignment and transcript assembly on the same set of 52 billion reads. Rockhopper required, on average, 25 minutes per file for a total time of 1457 hours. Rockhopper found the same rate, 76%, of reads aligning to the *E. coli* genome as that found by *HISAT2*. Transcripts identified by Rockhopper for all *E. coli* genes and their expression across the 3,376 samples are reported at https://dataverse.harvard.edu/api/access/datafile/6767650. When comparing transcript expression across the samples as determined via *StringTie* and via Rockhopper, we observed strong concordance. The median correlation across the samples between expression reported by the two tools was 0.993. Only 21 of the 3,376 samples had non-statistically significant *p*-values (*p* > 0.01) when determining correlation, and each of these 21 samples corresponded to datasets with less than 1% of the sequencing reads aligning to the *E. coli* genome.

### Expression

**Figure 2.**
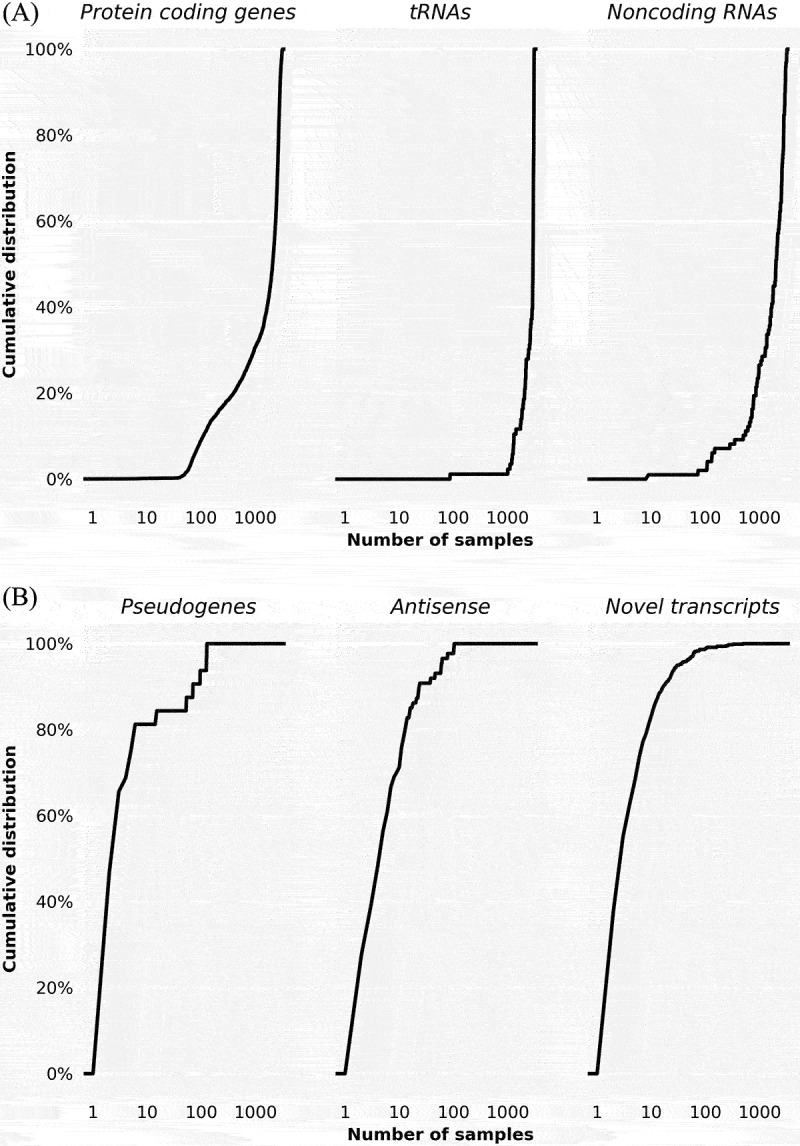
Cumulative distribution of the percentage of transcripts expressed in a given number of non-size-selected samples on a log scale. (A) Cumulative distributions for annotated *E. coli* genes corresponding to protein coding genes, tRNAs, and noncoding RNAs. (B) Cumulative distributions for transcripts corresponding to pseudogenes, antisense transcripts, and other novel transcripts that are not part of annotated *E. coli* genes.

For observed transcripts corresponding to annotated *E. coli* genes, we looked specifically at three families: protein coding genes, tRNAs, and annotated noncoding RNAs. For other observed transcripts, i.e. that do not correspond to annotated *E. coli* genes, we also looked at three families: pseudogenes, antisense transcripts, and novel transcripts. Figure 2 shows the cumulative distribution of how many transcripts in each of these families are expressed in the 3,325 non-size-selected samples. For observed transcripts corresponding to annotated genes (protein coding, tRNAs, or noncoding RNAs), the transcripts are generally expressed in many samples, e.g. more than 1,000 samples. For other observed transcripts (pseudogenes, antisense, novel transcripts), the transcripts are generally expressed in few samples, e.g. less than 100 samples. As points of reference, the 10 annotated genes that are expressed in the largest number of samples and the 10 annotated genes that are expressed in the smallest number of samples are shown in Table 2. Similarly, for the 51 size-selected samples, Supplementary Figure S1 shows the cumulative distribution of how many transcripts are expressed in each of the different families of transcripts. A major difference between expression in non-size-selected samples (Figure 2) and size-selected samples (Supplementary Figure S1) is that there is significantly less evidence of expression of transcripts corresponding to protein coding genes in the size-selected samples, consistent with the aim of size-selection experiments to minimize assays of longer transcripts, which generally correspond to protein coding genes.

**Table 2.** Ten annotated *E. coli* genes expressed in the most samples and ten annotated *E. coli* genes expressed in the fewest samples. The Type column indicates if the gene is annotated as a noncoding RNA (ncRNA), ribosomal RNA (rRNA), or protein coding gene (CDS).

Rank	Number of samples in which expressed	B#	Name	Type	Start Coord	Stop Coord	Strand	Product
1	3268	b2621	ssrA	ncRNA	2755593	2755955	+	tmRNA
2	3257	b3854	rrlA	rRNA	4037519	4040423	+	23S ribosomal RNA
3	3257	b3970	rrlB	rRNA	4168641	4171544	+	23S ribosomal RNA
4	3256	b4009	rrlE	rRNA	4210043	4212946	+	23S ribosomal RNA
5	3254	b3758	rrlC	rRNA	3943704	3946607	+	23S ribosomal RNA
6	3251	b1677	lpp	CDS	1757421	1757657	+	murein lipoprotein
7	3250	b3123	rnpB	ncRNA	3270216	3270592	-	RNase P catalytic RNA component
8	3248	b3851	rrsA	rRNA	4035531	4037072	+	16S ribosomal RNA
9	3247	b0204	rrlH	rRNA	225759	228662	+	23S ribosomal RNA
10	3247	b3968	rrsB	rRNA	4166659	4168200	+	16S ribosomal RNA
…								
4501	106	b2190	yejO	CDS	2286390	2288914	-	adhesin-like autotransporter YejO
4502	102	b4786	yqhJ	CDS	3111266	3111325	+	protein YqhJ
4503	69	b4668	ibsB	CDS	2153681	2153737	-	putative toxic peptide IbsB
4504	66	b4667	ibsA	CDS	2153349	2153408	-	toxic peptide IbsA
4505	61	b4714	ralA	ncRNA	1413556	1413734	+	small regulatory RNA antitoxin RalA
4506	48	b4570	lomR	CDS	1428796	1428984	+	Rac prophage; protein LomR_1
4507	13	b4571	wbbL	CDS	2101396	2101744	-	interrupted rhamnosyltransferase WbbL
4508	9	b0542	renD	CDS	568035	568247	+	protein RenD
4509	4	b3443	yrhA	CDS	3584205	3584309	+	putative uncharacterized protein YrhA
4510	2	b4569	yhcE	CDS	3366755	3366929	+	putative uncharacterized protein YhcE

**Figure 3.**
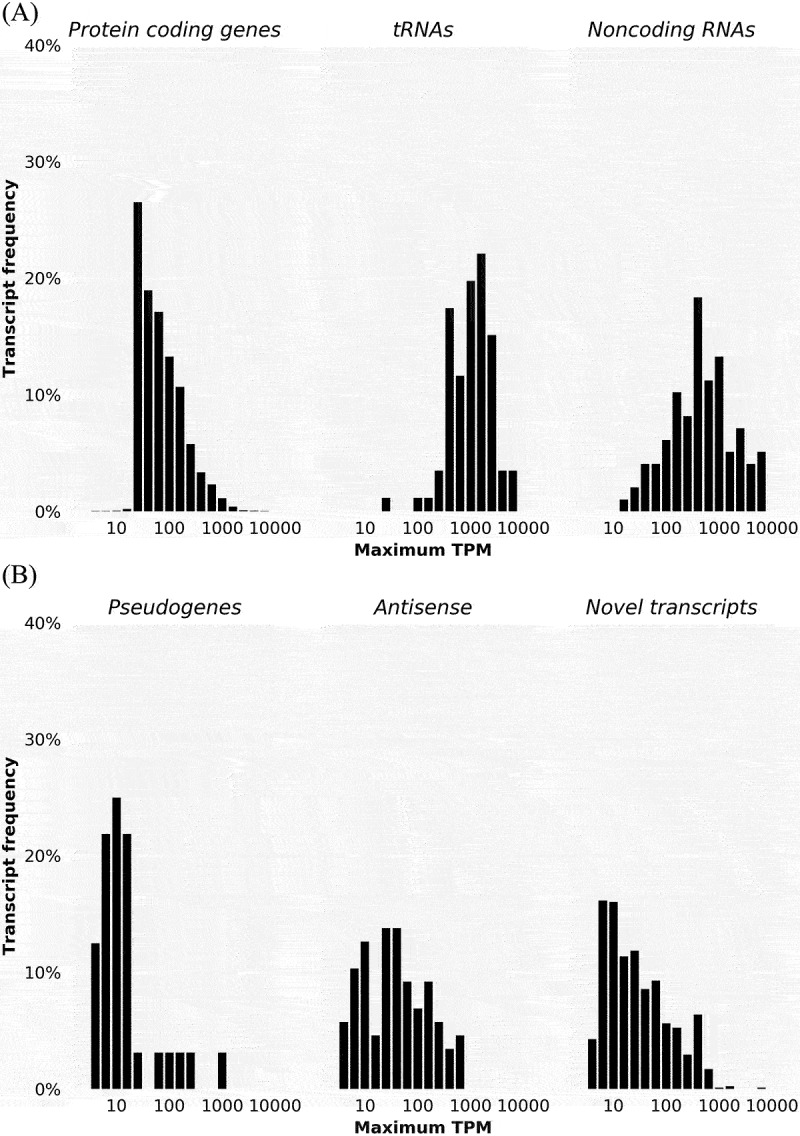
Frequency of transcripts expressed at different levels, as measured by the maximum TPM of a transcript across all non-size-selected samples on a log scale. (A) Frequency for annotated *E. coli* genes corresponding to protein coding genes, tRNAs, and noncoding RNAs. (B) Frequency for transcripts corresponding to pseudogenes, antisense transcripts, and other novel transcripts that are not part of annotated *E. coli* genes.

We also explored the expression level of transcripts as measured by TPM (transcripts per million). Figure 3 shows the percentage of transcripts expressed at different TPM levels for transcripts corresponding to annotated genes (protein coding genes, tRNAs, and noncoding RNAs) and for other transcripts (pseudogenes, antisense transcripts, and novel transcripts) in the 3,325 non-size-selected samples. Similarly, Supplementary Figure S2 shows the percentage of transcripts expressed at different TPM levels in the 51 size-selected samples. In general, transcripts corresponding to annotated genes are expressed at significantly higher levels than other transcripts.

In order to assess if gene expression profiles across the large number of RNA-seq experiments could provide information about genes’ roles in a cell, we used each gene’s expression profile to predict whether it corresponded to any of three Gene Ontology (GO) roles, namely, *molecular function*, *cellular component*, and *biological process* [22]. According to GO, *molecular function* refers to activities performed by gene products that occur at the molecular level, such as ‘catalysis’ or ‘transport’, *cellular component* refers to cellular anatomy and locations relative to cellular structures in which a gene product performs a function, such as cellular compartments or macromolecular complexes, and *biological process* refers to larger biological processes accomplished by multiple molecular activities, such as ‘DNA repair’ or ‘signal transduction’ [22]. In GO, genes may correspond to multiple roles. We used a multi-label logistic regression model to classify genes into these three roles based on their expression profile across the compendium. Using the gene expression profiles to predict GO roles, the logistic regression classifier had similar performance levels when identifying each of the three roles, as evaluated by six measures (Table 3 and Supplementary Figure S3). Based on these results, overall, we conclude that the expression profile of a gene can be used as a reasonably good, though not perfect, predictor of its GO role, though the high recall relative to precision of our classifier (Table 3) suggests that the approach we are using is, at times, over-predicting roles.

**Table 3.** Performance of a logistic regression algorithm for classifying genes into one of three Gene Ontology (GO) roles based on genes’ expression profiles across the RNA-seq samples. There are three GO roles, cellular component, molecular function, and biological process, and genes may correspond to one, two, or three of these roles. Performance is assessed by six measures: accuracy, precision, recall, F1 score, area under ROC curve, and area under precision-recall curve.

	Cellular Component	Molecular Function	Biological Process
Accuracy	75%	83%	86%
Precision	75%	84%	86%
Recall	100%	100%	100%
F1 score	86%	91%	93%
Area under ROC curve	70%	68%	66%
Area under PR curve	88%	91%	93%

### Transcription start sites

Differential RNA-seq (dRNA-seq) is an effective approach for identifying precise transcription start sites (TSSs) with single nucleotide resolution owing to its ability to distinguish primary and processed transcripts [23]. While traditional RNA-seq experiments such as those founding the compendium used in this study may not be as precise as dRNA-seq, they can still provide information about a transcript’s approximate start site. For example, one study found that TSSs identified from RNA-seq experiments with *E. coli* differed by 11 nucleotides, on average, from TSSs identified via 5’ RACE experiments, and TSSs identified from RNA-seq experiments with *N. gonorrhoeae* differed by 21 nucleotides, on average, from TSSs identified via primer extension analysis [21]. Here, we compared TSSs identified from the compendium with a set of previously published TSSs identified by dRNA-seq for 1,879 *E. coli* genes [24]. One challenge with such a comparison is that many different TSSs were identified for each gene across the range of RNA-seq experiments using our pipeline. The variety of TSSs identified for a given gene may be explained by the lack of precision in identifying transcription boundaries using RNA-seq data, by the existence of multiple alternative sites for transcription initiation in different experimental conditions, and by other factors. When considering the most commonly observed TSS for a gene across the 3,376 samples, this mode TSS differed from the dRNA-seq identified TSS by 18 nucleotides, on average. If we consider *any* TSS identified for a gene in the 3,376 samples rather than just the single most commonly observed TSS, we found that for 93% of the 1,879 genes, some TSS identified by the RNA-seq analysis corresponded precisely to the TSS identified by dRNA-seq.

### Transcription units

A transcription unit is a set of consecutive genes on the same strand of a genome that are co-transcribed into a single polycistronic message. We used our 3,376 transcript assemblies as input to train a logistic regression model in order to identify transcription units throughout the *E. coli* transcriptome [25]. Two sets of co-transcribed genes were considered. First, we looked at every pair of consecutive genes on the same strand of the genome to assess if the pair of genes was part of the same message. We found evidence for co-transcription of 2,067 pairs of genes and, for each pair of genes evincing co-transcription, we report the probability that the genes are co-transcribed as part of the same polycistronic message (https://dataverse.harvard.edu/api/access/datafile/6562496). Second, we looked for evidence of complete transcription units, i.e. sets of two or more genes that are all co-transcribed together into a single polycistronic message. We found evidence for co-transcription of 887 different multi-gene transcription units encompassing 2,954 genes (https://dataverse.harvard.edu/api/access/datafile/6562497). To evaluate these transcription unit identifications, we compared them to RegulonDB [26], a database of experimentally confirmed operons in *E. coli*, and we observed a sensitivity of 94%, indicating that the transcription units observed in our transcript assemblies are well aligned with previously documented operons. Further, we observed co-transcription evidence for 458 pairs of genes not identified in the RegulonDB database, suggesting potential novel instances of polycistronic messages in *E. coli* (https://dataverse.harvard.edu/api/access/datafile/6562496). To further evaluate the co-transcription we observed in our transcript assemblies, we compared our transcription unit identifications to operon predictions from the Database of prOkaryotic OpeRons (DOOR^2^), a leading database of computationally predicted operons in bacteria [27]. We found the similarity, as measured by the RAND coefficient [28], between our transcription unit identifications and DOOR’s predictions to be 96%, indicating high concordance.

## Discussion

We designed, implemented, and evaluated a genome-guided assembly pipeline to investigate the transcriptome of *E. coli* K-12 MG1655. We applied the pipeline to a compendium of thousands of RNA-seq samples from the Sequence Read Archive. The 24 terabytes of sequencing data used here consist of 52 billion reads and more than 7 trillion sequenced bases. Based on this rich dataset, we generated thousands of condition specific transcript assemblies. When we merged the assemblies, we found evidence of transcription for every annotated *E. coli* gene as well as for 5,071 other transcripts corresponding, for example, to pseudogenes, antisense transcripts, and candidate novel regulatory RNA or other small genes. Altogether, transcripts corresponding to annotated genes had significantly higher expression levels and were expressed in a significantly larger number of conditions than other transcripts.

The more than five thousand transcripts that we observed beyond annotated genes warrant more careful investigation to understand whether they correspond to genes with functional transcripts as opposed to pervasive transcription or transcriptional noise, so that the false-discovery rate can be better quantified [29]. Given the prevalence of small regulatory RNAs [30] and small proteins [31] in bacteria, further interrogation of our evinced unannotated transcripts will be important to ascertain to what extent they contain novel genes with functional implications. Similarly, widespread antisense transcription in bacteria has been well documented [32], with one thorough analysis finding that 37% of all transcription start sites in *E. coli* correspond to antisense transcripts [24], consistent with the antisense transcription we observe in this study when analysing our compendium of RNA-seq data sets.

Our transcript assemblies allowed us to investigate the landscape of multi-gene transcription units employed in *E. coli*, as well. We observed evidence for nearly three thousand *E. coli* genes being transcribed as part of polycistronic messages. The transcription units we identified have high correspondence with previously documented operons. We also found evidence of co-transcription for 458 pairs of genes not reported in a leading operon database. As genes that are co-transcribed often have related functional roles or participate in the same metabolic pathways, characterizing multi-gene transcription units can help illuminate the functional relationships of genes and co-regulation [33].

By using such a large compendium of sequencing data, we have comprehensively characterized the *E. coli* transcriptome. We hope that our results, indicating to what extent every gene is expressed in each of thousands of conditions, will serve as a resource to the community. For researchers interested in bacteria beyond *E. coli*, our results provide guidance in regard to how many RNA-seq data sets are necessary to observe the full range of gene expression and capture the complete transcriptome of the bacteria, assuming the samples for a different bacteria represent a similar distribution of conditions with analogous levels of gene expression as the samples we used for *E. coli*. Finally, the thousands of transcripts we observe that do not correspond to annotated genes demonstrate the complexity of bacterial RNA biology, in that even one of the most studied and best understood organisms has considerable transcription activity that we have not yet fully elucidated.

## Materials and methods

### Data acquisition

The *Escherichia coli* K-12 MG1655 genome (assembly ASM584v2) and annotation were downloaded from RefSeq [34]. Accession numbers for sequencing read samples were downloaded from the Sequence Read Archive (SRA) [1]. The Entrez Programming Utilities (E-utilities) [35] tools *esearch* and *efetch* were used to download detailed data associated with each sequencing read sample, including information about the run, release date, spots, bases, read length, size, download path, experiment, library information, platform, BioProject, BioSample, and other information. Samples were filtered to identify those with an RNA-seq library strategy and a transcriptomic library source. There were 3,376 samples that met the filtering criteria. FASTQ sequencing read files were downloaded for the 3,376 samples using the SRA Toolkit version 3.0.0 tools *prefetch* and *fasterq-dump* [36].

### Alignment and assembly

Sequencing reads from FASTQ files were aligned to the *E. coli* genome using HISAT2 version 2.2.1 to produce a SAM file for each sample [37]. Default parameter settings for HISAT2 were used (–n-ceil L,0,0.15 –mp 6,2 –sp 2,1 –np 1 –rdg 5,3 –rfg 5,3 –score-min L,0.0,-0.2 -k 5 –max-seeds 10). SAM files were sorted and converted to binary BAM format using SAMtools version 1.15.1 [38]. Aligned and sorted reads were assembled using StringTie version 2.2.1 (parameters -m 50 -s 1 -c 1 -g 5) to produce a GFF file for each sample [39]. GffCompare version 0.12.6 was used to merge assemblies into a single combined GFF file [40]. Rockhopper version 2.0.3 was used for alignment, assembly, and operon identification using default parameter settings (-d 500 -a true -m 0.15 -l 0.33 -y true -t true -z 0.5) [21,25].

### Machine learning methods

Prior to applying the machine learning algorithm, data were scaled so that each feature had a mean of zero and a standard deviation of one. A logistic regression model was then trained as a supervised classification approach with a limited-memory BFGS optimization algorithm and an L2 penalty term. Ten-fold cross-validation was used to tune the regularization hyperparameter for the logistic regression model. For implementation of the machine learning methods, the Scikit-learn library linear_model.LogisticRegression was used [41].

## Supplementary Material

Supplemental Material

## Data Availability

The data that support the findings of this study, including all transcript assemblies and source code, are openly available in the Harvard Dataverse at https://doi.org/10.7910/DVN/QBMC9D. All source code is publicly available on GitHub at https://github.com/btjaden/Compendium.git.
