# Supplementary figures and images for: Escherichia coli transcriptome assembly from a compendium of RNA-seq data sets
Source: RNA Biol. 2023 Mar 15;20(1):77–84. doi: 10.1080/15476286.2023.2189331 (PMC10392735; doi:10.1080/15476286.2023.2189331)

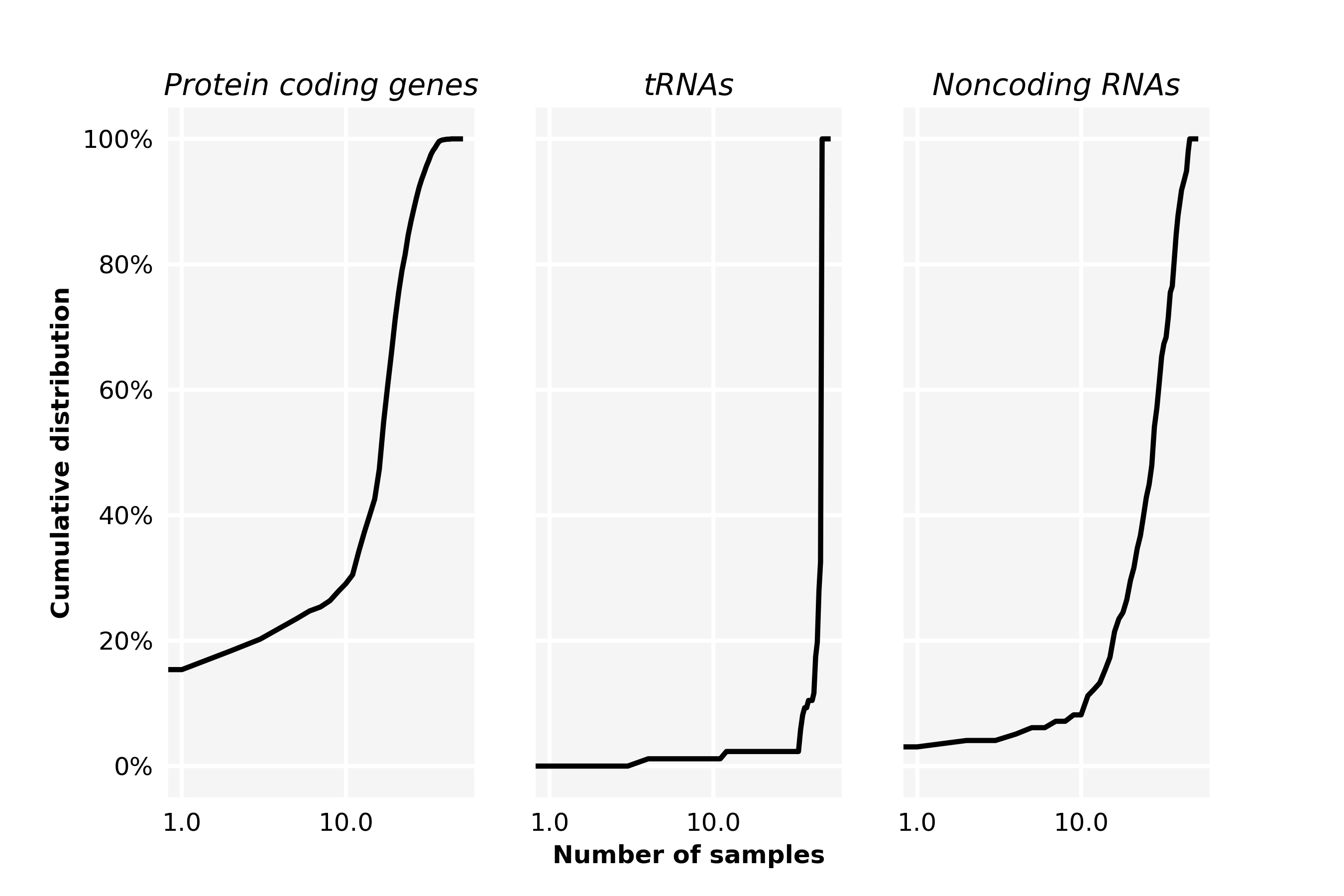

Supplement: Supplemental Material [file KRNB_A_2189331_SM7113.zip › Supp_2189331 (1)/Supp_2189331/Supp_Figure1a (1).jpg]

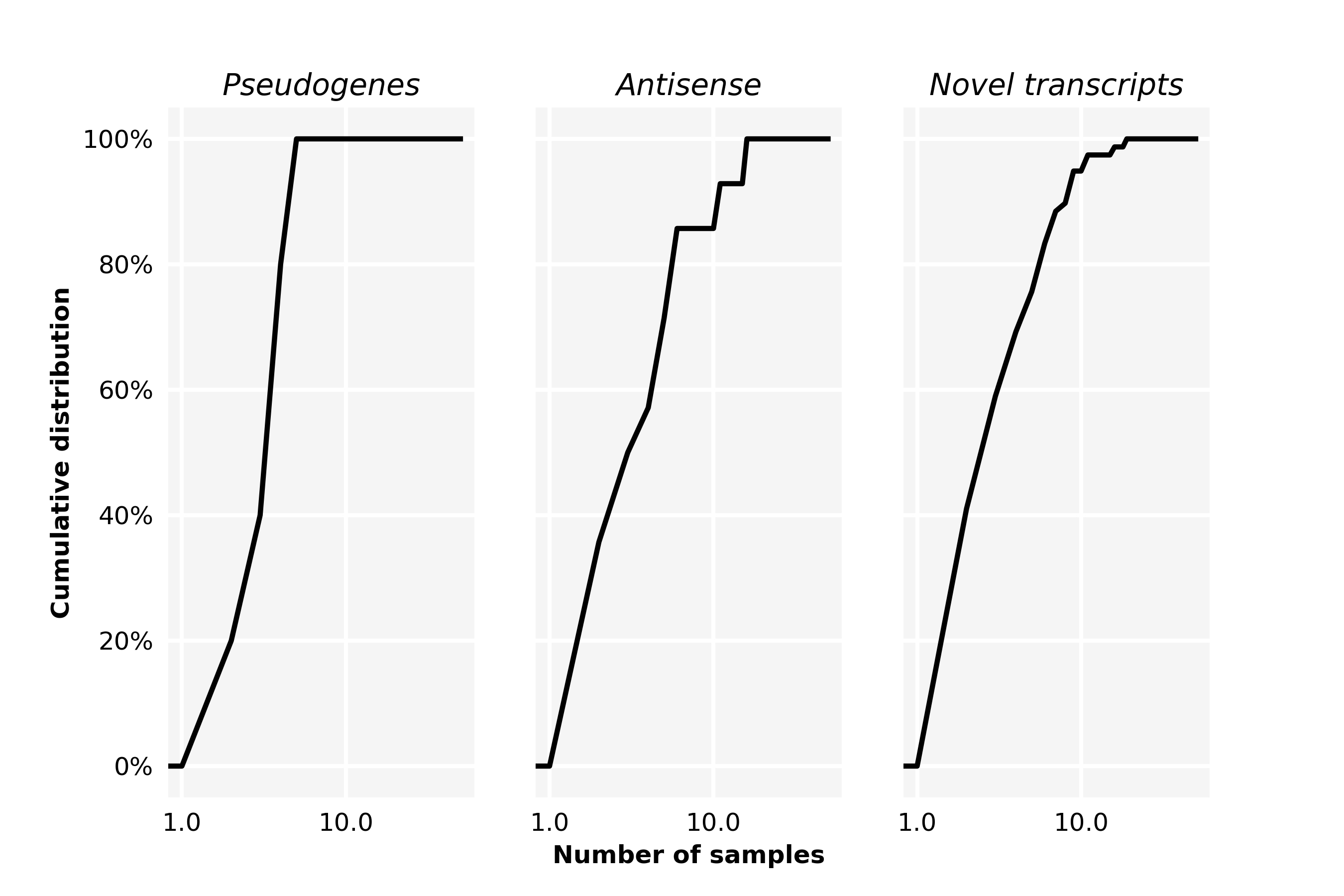

Supplement: Supplemental Material [file KRNB_A_2189331_SM7113.zip › Supp_2189331 (1)/Supp_2189331/Supp_Figure1b.jpg]

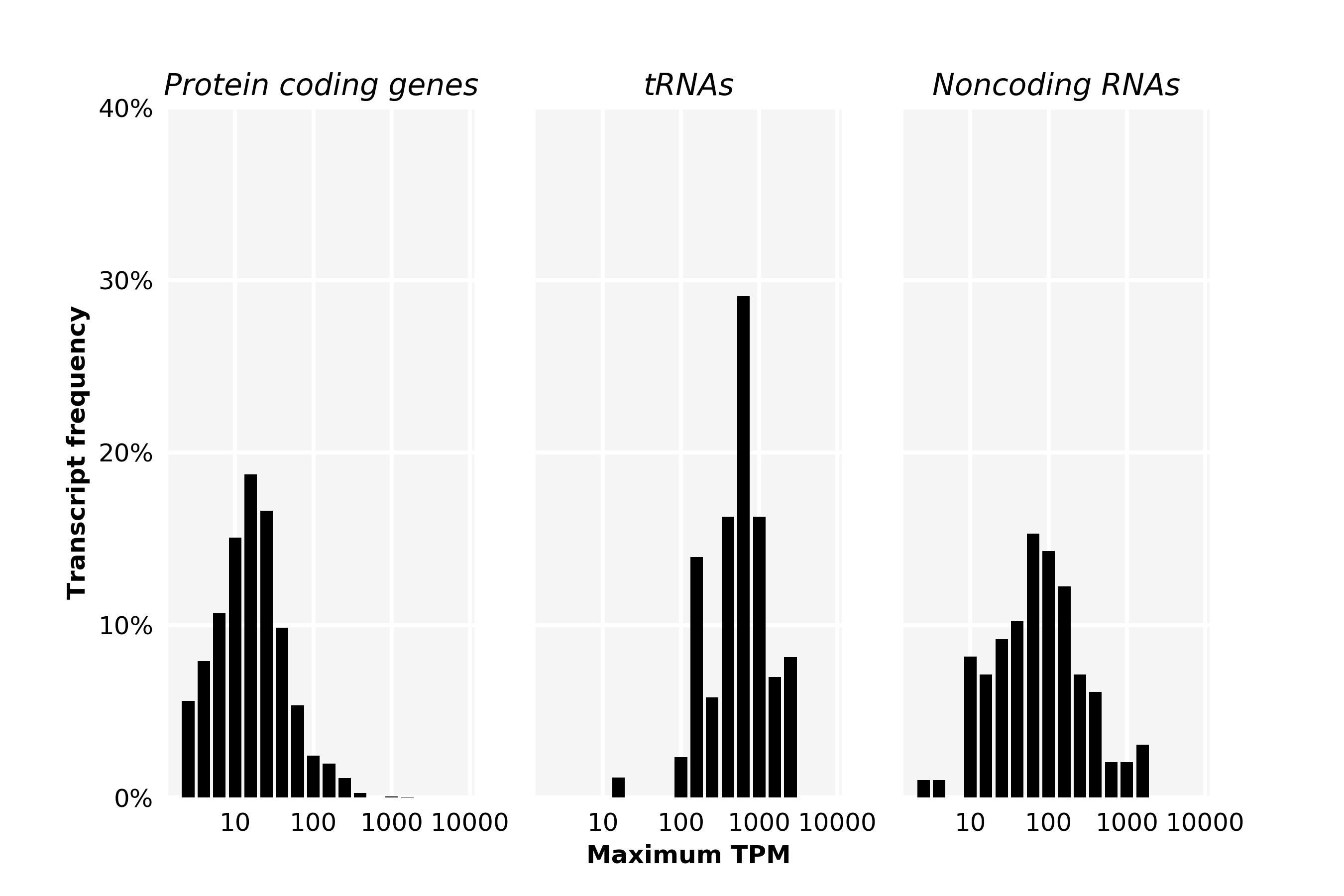

Supplement: Supplemental Material [file KRNB_A_2189331_SM7113.zip › Supp_2189331 (1)/Supp_2189331/Supp_Figure2a.jpg]

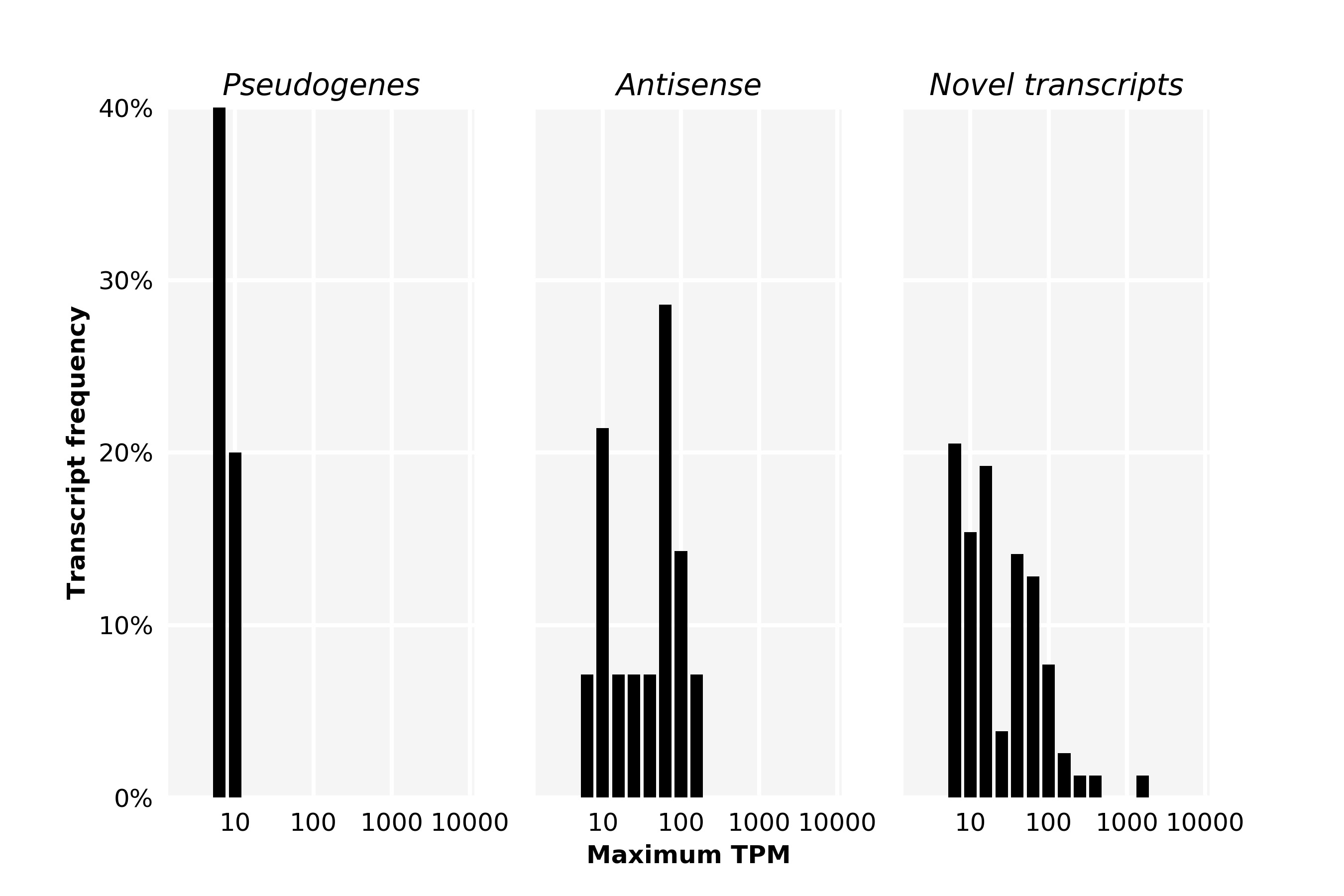

Supplement: Supplemental Material [file KRNB_A_2189331_SM7113.zip › Supp_2189331 (1)/Supp_2189331/Supp_Figure2b.jpg]

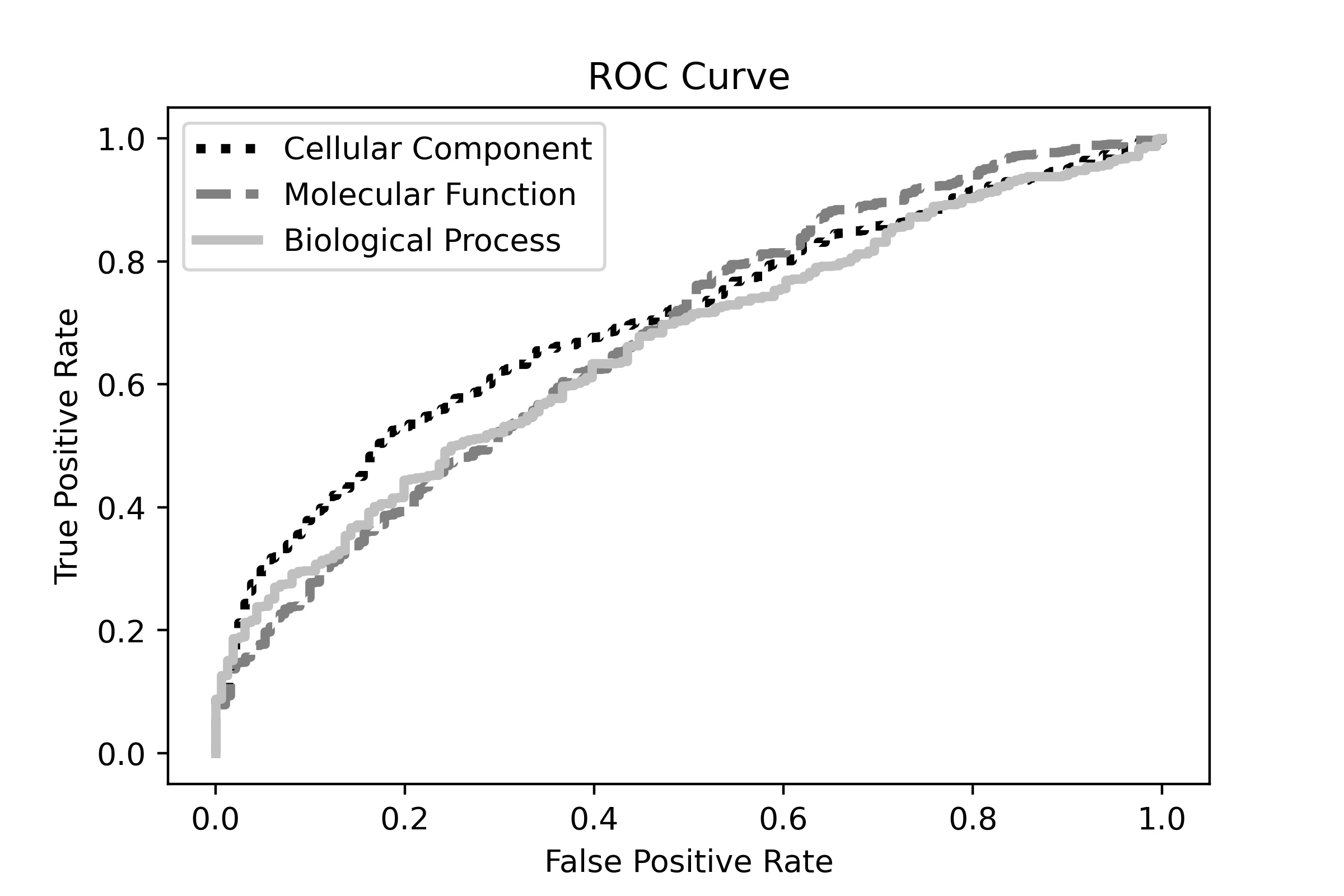

Supplement: Supplemental Material [file KRNB_A_2189331_SM7113.zip › Supp_2189331 (1)/Supp_2189331/Supp_Figure3.jpg]
